# Associations between the Complement System and Choroidal Neovascularization in Wet Age-Related Macular Degeneration

**DOI:** 10.3390/ijms21249752

**Published:** 2020-12-21

**Authors:** Emilie Grarup Jensen, Thomas Stax Jakobsen, Steffen Thiel, Anne Louise Askou, Thomas J. Corydon

**Affiliations:** 1 Department of Biomedicine, Aarhus University, 8000 Aarhus C, Denmark; egj@biomed.au.dk (E.G.J.); st@biomed.au.dk (S.T.); ala@biomed.au.dk (A.L.A.); 2 Department of Ophthalmology, Aarhus University Hospital, 8200 Aarhus N, Denmark; thomasstaxjakobsen@gmail.com

**Keywords:** age-related macular degeneration, complement system, choroidal neovascularization, anti-complement therapy

## Abstract

Age-related macular degeneration (AMD) is the leading cause of blindness affecting the elderly in the Western world. The most severe form of AMD, wet AMD (wAMD), is characterized by choroidal neovascularization (CNV) and acute vision loss. The current treatment for these patients comprises monthly intravitreal injections of anti-vascular endothelial growth factor (VEGF) antibodies, but this treatment is expensive, uncomfortable for the patient, and only effective in some individuals. AMD is a complex disease that has strong associations with the complement system. All three initiating complement pathways may be relevant in CNV formation, but most evidence indicates a major role for the alternative pathway (AP) and for the terminal complement complex, as well as certain complement peptides generated upon complement activation. Since the complement system is associated with AMD and CNV, a complement inhibitor may be a therapeutic option for patients with wAMD. The aim of this review is to (i) reflect on the possible complement targets in the context of wAMD pathology, (ii) investigate the results of prior clinical trials with complement inhibitors for wAMD patients, and (iii) outline important considerations when developing a future strategy for the treatment of wAMD.

## 1. Introduction

**Figure 1:**
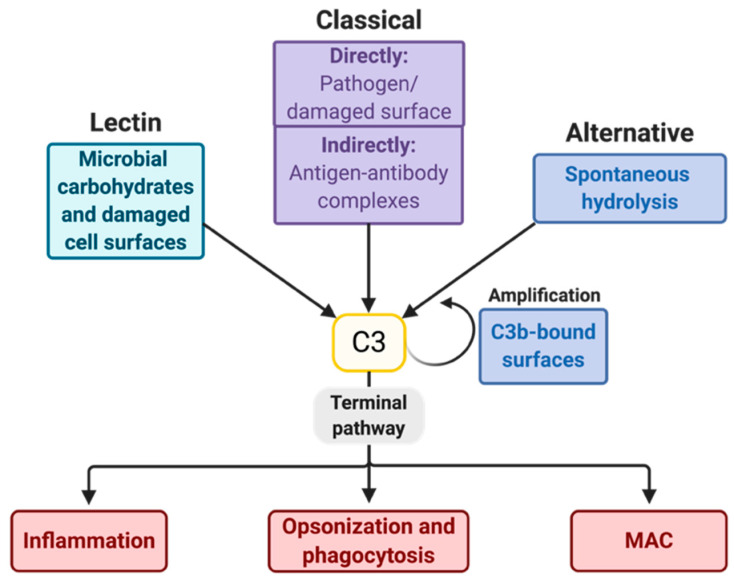
Pathways of the complement system. Many surfaces of pathogens, immune complexes, or modified host surfaces (e.g., necrotic and apoptotic cells [10,11]) activate the complement system. In the lectin pathway (LP), microbial carbohydrates or modified self surfaces [12] are recognized by lectins, either by one of the two collectins, mannose-binding lectin (MBL) or collectin-LK, or by one of three ficolins [13]. C1q is the recognition component of the classical pathway (CP) and recognizes either deposited immunoglobulins (Igs) or C-reactive protein [14], or binds the pathogen or apoptotic cell directly [10]. Complement component 3 (C3) may also be activated through spontaneous hydrolysis of its thioester in the alternative pathway (AP). The AP can also amplify C3-responses via the formation of a C3-convertase. The cleavage of C3 initiates the terminal pathway (TP), which introduces inflammation, opsonization, and subsequent phagocytosis as well as the formation of the membrane-attack complex (MAC). The MAC stimulates certain signaling pathways [15,16,17,18] or lyses cells to disturb their integrity. AP, alternative pathway. C3, complement component 3. CP, classical pathway. Ig, immunoglobulin. LP, lectin pathway. MAC, membrane-attack complex. TP, terminal pathway.

Age-related macular degeneration (AMD) is a multi-factorial retinal disease with a significant inflammatory contribution, which is presently being explored. Several recent findings have strongly associated AMD with the complement system [1,2,3,4,5,6,7,8,9], thereby pinpointing the pivotal role of complement factors in the development of AMD. The complement system is a part of the innate immune system and is activated by either pathogens or damaged host cells. The initiating pathways of complement activation are the lectin pathway (LP), classical pathway (CP), and alternative pathway (AP), which all revolve around the cleavage of complement component 3 (C3) (Figure 1). It is still uncertain which pathways are involved in the pathophysiology of wet AMD (wAMD). However, the amplification ability of the AP seems to be crucial. Since the retina has high metabolic demands, its tissue is particularly vulnerable to oxidative damage, and a small local injury may be sufficient for amplification of the complement response and the development of AMD.

**Figure 2:**
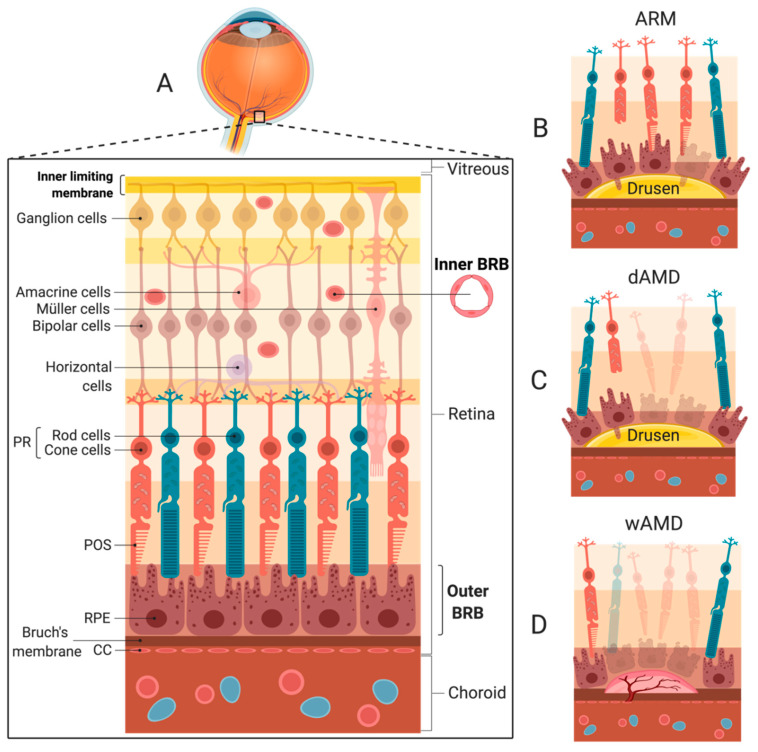
The cellular organization of the retina, age-related maculopathy (ARM), and age-related macular degeneration (AMD) stages. (**A**) The different cell layers of the retina are depicted spanning from the vitreous body to the choroid. The outer layer of the retina comprises photoreceptor (PR) cells and the retina pigment epithelium (RPE). Bruch’s membrane stretches from the plasma membrane of the RPE to the choriocapillaris (CC) in the choroid, which is the vascularized layer of the eye comprising vessels and connective tissue. The inner limiting membrane is the boundary between the retina and the vitreous formed by Müller cell footplates. (**B**) Progression of the healthy retina to age-related maculopathy (ARM) is ascertained by the presence of drusen and/or abnormal pigmentation. (**C**) Geographic atrophy (GA) is distinguished by one or more sharply delineated areas of at least 175 µm in diameter with abnormal pigmentation or local areas of complete RPE atrophy and more visible choroidal vessels than the surrounding areas. (**D**) wet AMD (wAMD) is ascertained when RPE detachment and/or choroidal neovascularization (CNV) is present. Acute vision loss can be experienced due to the accumulation of subretinal and/or intraretinal fluid with a loss of structural and functional retinal integrity. ARM, age-related maculopathy. BRB, blood-retina barrier. CC, choriocapillaris. CNV, choroidal neovascularization. dAMD, dry AMD. GA, geographic atrophy. POS, photoreceptor outer segment. PR, photoreceptor. RPE, retinal pigment epithelium. wAMD, wet AMD.

AMD is the leading cause of age-related blindness in the Western world [19]. AMD affects ~10% of Europeans above the age of 85 [20] and 1.7% of Dutch individuals between the ages of 55 and 98 [21]. The prevalence of AMD is predicted to increase worldwide because of a shift toward aging populations [22]. AMD is characterized by progressive degeneration of the central retina, known as the macula, which is dominated by cone cells mediating high-resolution photopic vision and color discrimination. Consequently, AMD has a debilitating effect on visual function. The retinal pigment epithelium (RPE) and photoreceptor (PR) cells are especially affected in AMD. In the healthy outer retina, a symbiotic relationship is present between the PR cells, the RPE, Bruch’s membrane, and the choriocapillaris (CC) (Figure 2A). Components such as nutrients and waste are systematically transported across the polarized RPE to support the PR cells [23]. The RPE both nourishes the PRs and acts as phagocytic cells to maintain the integrity of these cells [24,25].

Different classifications are available for the stages of developing AMD [26,27,28,29]. In this review, we classify the disease stages based on the international classification and grading system for age-related maculopathy and AMD from 1995 [26], re-evaluated in 2003 [30], to distinguish the early age-related maculopathy (ARM) stages (Figure 2B) from the late and more severe ARM stage involving degeneration, i.e., AMD. The progression of a healthy retina to ARM is ascertained by the presence of drusen, i.e., deposits of waste, and/or abnormal pigmentation. Several distinct processes can be observed in the environment of drusen. However, their order is uncertain. RPE blebs can reach into early drusen [31]. Photoreceptor outer segments (POS) may be missing in the PR layer [32] and cells of the RPE, PR cells, and CCs are generally found to be dysfunctional or degenerated in the vicinity of drusen (Figure 2B).

AMD is characterized by noticeable vision loss, and one or both of the following presentations: geographic atrophy (GA) (dry AMD (dAMD)) (Figure 2C) or wAMD (also known as exudative or neovascular AMD) (Figure 2D). In patients suffering from wAMD, neovascular vessels from the CC known as choroidal neovascularizations (CNVs) develop beneath the RPE or penetrate into the subretinal space. Leakage from the immature neovascular vessels causes fluid extravasation, which is often accompanied by an acute loss of central vision due to macular edema as opposed to the slower progression of dAMD, for which vision loss is gradual over several years [33]. Currently, no treatment is available to halt ARM or dAMD, and the anti-angiogenic treatment available for wAMD is unpleasant, expensive, and has a risk of adverse effects [34,35].

Importantly, several clues indicate the essential role of the complement system in the pathogenesis of ARM and AMD [1,2,3,4,5,6,7,8,9]: (i) the presence of complement components in drusen, such as the complement peptide C3a and factor H (FH) [1,2,36,37,38,39,40], (ii) single nucleotide polymorphisms (SNPs) in genes encoding components and regulators of the complement system are associated with an increased risk of ARM and AMD [41], (iii) higher levels of the terminal complement complex (the membrane attack complex, MAC) in the retinas of ARM and AMD patients [42], especially in those with an FH risk variant [1,43,44], (iv) fewer or dislocated regulatory complement proteins in the eyes of ARM and AMD patients [45,46], (v) increased levels of systemic and active complement proteins in AMD patients compared to the controls [47,48,49,50], and (vi) elevated levels of local complement proteins in aqueous humor samples and the vitreous humor of AMD patients compared to the controls [51,52,53]. A recent genome-wide association study (GWAS) showed that multiple complement variants affect systemic complement activation. However, only some of these variants are associated with AMD and local disease in the retina, suggesting tissue-specific effects [41]. These findings have led to the hypothesis that AMD is a local manifestation of chronic low-grade systemic complement activation in aging patients with risk variants [49].

Several clinical trials with complement inhibitors have been initiated during the last decade based on the association between AMD and the complement system. Unfortunately, the results have, so far, not been successful. In this review, we focus on the association between the complement system and the pathology of wAMD and suggest that the choice of the complement factor target, the stage of AMD when treatment is initiated, and the route of administration are vital for success. Moreover, we consider the origin of the faulty complement stimulation by either systemic or locally produced activators, as well as the importance of choosing a suitable preclinical model. Finally, we focus on the wAMD trials and their complement protein targets to determine the trajectory of future therapeutic approaches.

## 2. The Complement System and Potential Targets for Inhibition

The complement system consists of ~50 soluble proteins [54] circulating in the blood as inactive components. When a triggering cleavage event happens, a cascade of proteases is commenced on a given surface on either the pathogens or host cells. The complement factors are primarily made by the liver [55], and some are produced as a part of the acute phase response. Others are produced by a wide range of different cell types [56], e.g., cells of the immune system [57,58] and cells in the retina [59].

**Figure 3:**
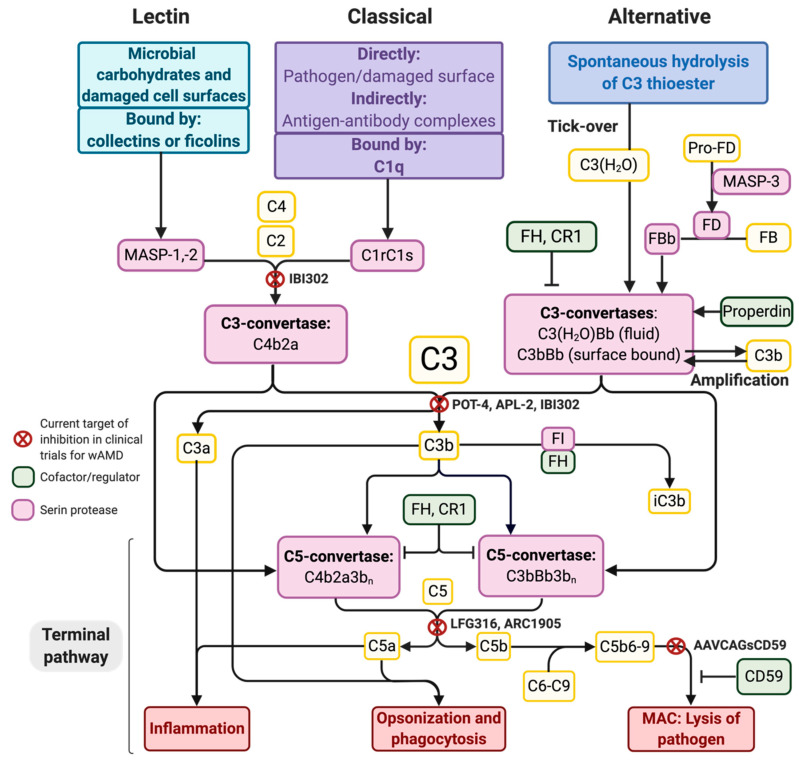
Overview of the complement components, regulators, and potential targets for inhibition. Selected complement components are depicted. All components except for MBL-associated serine protease-3 (MASP-3) [63] circulate in the blood as inactive components. Both the LP and the CP lead to the formation of C4b2a, a C3-convertase, while the alternative pathway (AP) comprises both a fluid-phase C3-convertase (C3(H_2_O)Bb) and a surface-bound C3-convertase (C3bBb). The fluid-phase convertase is made when C3 is spontaneously hydrolyzed in plasma and binds factor B (FB), which is then cleaved by factor D (FD), while the surface-bound convertase requires preformed C3b on a surface that will bind FB, which is cleaved to FBb by FD. Properdin is a stabilizing regulator for the C3-convertases [64,65,66]. C3(H_2_O) and C3b can be degraded into inactivated iC3(H_2_O) and iC3b, respectively, by the enzyme factor I (FI) and a co-factor, such as factor H (FH). The targets of inhibition in wAMD clinical trials are indicated. AP, alternative pathway. CR1, complement receptor 1. FB, factor B. FD, factor D. FH, factor H. FI, factor I. MAC, membrane-attack complex. MASP, MBL-associated serine protease.

As depicted in Figure 1, the LP, CP, and AP are initiative pathways that result in the cleavage of C3 and the subsequent induction of the terminal pathway (TP). The LP and CP mainly differ in their components that activate the pathways with an otherwise similar cleavage of C3 via the generation of the C4b2a complex, which is a C3 convertase (Figure 3). After C3 is cleaved into C3a and C3b, the TP is initiated, which involves three main events: inflammation, opsonization, and formation of the MAC (Figure 3). The complement peptides C3a, C4a, and C5a, called anaphylatoxins, appear when C3, C4, and C5 are cleaved, respectively. C3a and C5a especially stimulate inflammation and recruit and activate immune cells [60,61,62], e.g., by inducing degranulation of the mast cells [60]. C4a is a less efficient anaphylatoxin. Likewise, C4b, which is made when C4 is cleaved, is not as efficient as C3b in opsonizing pathogens in favor of phagocytosis. The production of C3b induces the formation of C5-convertases, which cleave C5 to C5a and C5b. C5b binds C6, C7, C8, and C9 to form MAC, which is a pore that can merge into the membrane onto which the components are recruited, leading to cell lysis and death (Figure 3).

The initiation of the alternative pathway (AP) deviates from the other two pathways, as C3 is also a subject of autoactivation through spontaneous hydrolysis (called tick-over), yielding C3(H_2_O) (Figure 3). Meanwhile, MBL-associated serine protease (MASP) 3 cleaves pro-Factor D into Factor D (FD) [63], allowing FD to cleave Factor B (FB) into the active fragment FBb (Figure 3). As a result, the fluid-phase C3-convertase, C3(H_2_O)Bb, may form. In this way, the AP is constitutively active to some degree, and the complement system is primed. A possibly more important function of the AP is that it can amplify the C3-responses initiated by any of the pathways, as factor B can bind deposited C3b and then become the active enzyme FBb after being cleaved by FD, thereby forming the membrane-bound C3-convertase, C3bBb (Figure 3).

The complement system is tightly regulated to prevent excessive activation and a resulting inflammatory reaction. Several regulatory proteins are present on host cells to induce, e.g., the dissociation of convertases and to stimulate C3b-degradation. Due to the ability of the AP to autoactivate, the control of this pathway is based on the constant degradation of C3 convertases. One of the main co-factors for C3-convertase degradation is soluble FH, which irreversibly dissociates C3-convertases with the help of factor I (FI). FH binds C3b-deposited surfaces and may simultaneously bind nearby polyanionic components (e.g., sialic acid on host membranes). FH also has an affinity for the glycosaminoglycans (GAGs) found in the extracellular matrix (ECM) [67]. Surfaces that are unable to, or inefficiently, bind complement-inhibiting proteins, such as FH, are not able to degrade C3-convertases, and the AP will be active. If sufficient C3bBb or C4b2a accumulates on a surface, it can generate extensive C3b and induce the formation of C5-convertases and the membrane attack complex (MAC) (Figure 3). If only sublytic levels accumulate, MAC can stimulate the signaling pathways associated with tissue remodeling, inflammation, and angiogenesis [15,16,17,18]. Moreover, MAC can lyse cells if accumulated at higher levels. Lysis is unwanted for host cells, even when diseased and degenerating. Thus, another role for FH when binding to necrotic and apoptotic cells [68] is to enable the FI cleavage of active C3b to form iC3b (Figure 3). This inactive component is used for the non-inflammatory removal of these cells. Furthermore, CD59 is a membrane-bound protector of healthy cells binding to C8 and preventing the formation of MAC and, thus, cell lysis (Figure 3).

## 3. Genetic Associations between AMD and the Complement System

Several genetic linkage analyses in large family-based studies [69,70,71,72,73] and subsequent GWAS focusing on AMD [74,75,76,77,78,79] established the significant associations between AMD and specific genomic regions, particularly in chromosomes 1 and 10. Genetic polymorphisms in two specific genomic regions were found to be major risk factors for developing AMD: 1q32 comprising the *FH* gene and 10q26 comprising the *ARMS2*/*HTRA1* gene [75,80]. Subsequently, genetic variation in other complement genes was associated with AMD development and progression [81], such as the genetic variants found in the genes *C3* [82], *FI* [83], *C2/FB*, *FH*-related genes [4], and *C9* [83]. The many variants found in the genes related to the complement system [4,9,80,81,84] highlight the importance of this immunologic pathway in AMD etiology.

The Y402H mutation in *FH* (rs1061170) is the most strongly associated common risk variant of ARM and AMD [81]. The SNP was found to yield an increased likelihood of developing wAMD of 1.9–2.34 per allele [85], and homozygous individuals were found to have an increased likelihood of 5.78 [86]. This may be due to the general protecting role of FH in the retina, such as its ability to decrease the complement activation on host cells and exert anti-oxidant effects towards the RPE [87] as well as its critical role in extracellular compartments with no intrinsic expression of regulators against the AP. In AMD, Bruch’s membrane is a specialized extracellular component at risk. With the expression of the Y402H allele, the binding of the FH Y402H to GAGs in Bruch’s membrane is impaired [45,67,88,89], which leaves some individuals more prone to AMD — e.g., deposited MAC is found to be increased by >60% in the choroids of 402H homozygous individuals compared to 402Y homozygous individuals [43]. Moreover, FH Y402H binds poorly to surfaces coated with the oxidative stress marker malondialdehyde [88] (e.g., apoptotic cells [90]), leaving these cells tagged for inflammatory degradation. The Y402H variant, along with risk alleles of the HTRA1/ARMS2 region, is highly associated with wAMD [86]. However, a study found that *FH* risk alleles are generally more strongly associated with dAMD [75].

A nonsense mutation in *C9* (rs121909592) holds a 4.7-reduced likelihood of developing wAMD [91], substantiating the role of the MAC in AMD. R32Q (rs641153) and R32W (rs12614) are variants of *FB* associated with protection against wAMD, as R32Q and R32W FB relate to reduced binding to C3b. Moreover, an SNP (R102G, rs2230199) in the gene of *C3* was associated with an increased risk of AMD [82], which may be accounted for by a decreased binding affinity of C3b toward FH [81]. This may yield reduced FH-dependent degradation of C3b, an extended lifetime of the convertases, and, consequently, enhanced AP activation. Together, these findings suggest that genetic variants that hinder the negative regulation of the complement system promote AMD development and that the variants inhibiting activation are protective.

## 4. Choroidal Neovascularization and wAMD

The hallmark of wAMD is the formation of a choroidal neovascular membrane. This CNV formation consists of neovascular vessels sprouting from the CCs into avascular spaces in or beneath the retina (Figure 2D) [92]. These immature vessels are leaky, causing fluid extravasation with the formation of intra-retinal or sub-retinal edema, as well as RPE detachments. The edema is often accompanied by hemorrhages and lipid deposits known as exudates. Rather than being a specific feature of AMD, this phenomenon can be considered a stereotypical response to different pathological stimuli and complicates several chorioretinal disorders, such as AMD, high myopia, and posterior uveitis [93].

**Figure 4:**
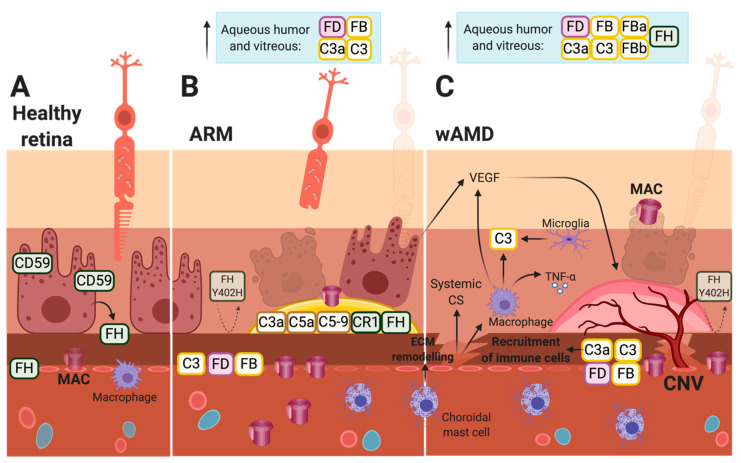
Patient samples reveal increased complement deposition in ARM and AMD eyes. (**A**) The healthy human retina is protected from the complement system by Factor H and CD59, and potential inflammatory immune cells are localized on the choroidal side of the outer blood–retina barrier (BRB). (**B**,**C**) Complement components are deposited in different compartments of the eyes in both ARM and wAMD patients. Loyet et al. found the highest level of complement deposition in the CC followed by Bruch’s membrane [52]. The RPE, macrophages, and complement system are involved in the production of vascular endothelial growth factor (VEGF) and, thus, the formation of CNV. AP, alternative pathway. ARM, age-related maculopathy. BRB, blood–retina barrier. CC, choriocapillaris. CNV, choroidal neovascularization. CP, classical pathway. ECM, extracellular matrix. FB, Factor B. FD, Factor D. FH, Factor H. FI, Factor I. LP, lectin pathway. MAC, membrane-attack complex. PR, photoreceptor. RPE, retinal pigment epithelium. TNF-α, tumor necrosis factor-alpha. VEGF, vascular endothelial growth factor.

The pathogenesis of CNV formation in wAMD is not fully elaborated but involves a dynamic process of inflammation, angiogenesis, and proteolysis with remodeling of the ECM [94,95]. It is possible to divide CNV development into stages of initiation, maturation, and involution [96]. Pathological changes in Bruch’s membrane combined with pro-angiogenic and inflammatory factors enable the invasion of endothelial cells, pericytes, fibrocytes, and inflammatory cells into the sub-RPE or subretinal space [92]. In the following active inflammatory phase, several mediators are produced by, e.g., the RPE, retinal glial cells (Müller cells and microglia), endothelial cells, and invading macrophages [96]. Important pro-angiogenic factors include vascular endothelial growth factor (VEGF), a key player in CNV [97,98,99,100,101], as well as the platelet-derived growth factor and basic fibroblast growth factor, while angiostatic proteins include pigment endothelial-derived factor (PEDF), angiostatin, and endostatin [94]. In the retina, VEGF is mainly produced by the RPE along with the anti-angiogenic PEDF. Thus, the RPE plays an important role in angiogenic homeostasis beneath the retina [102,103]. The production of VEGF can be modulated not only by the level of oxygen [104] but also by the presence of insulin-like growth factor I [105], glucose [104], reactive oxygen intermediates [106], and complement components [15,107,108]. Moreover, macrophage infiltration is a known feature of AMD histopathology [109], and these immune cells can be regarded as a relevant source of VEGF and other important cytokines, such as tumor necrosis factor-alpha (TNF-α) [110]. Moreover, resident choroidal mast cells are present in a significantly increased number in ARM and AMD eyes compared to age-matched controls, and their degranulation of proteolytic enzymes could be associated with the pathogenesis of AMD, as degranulated mast cells are found close to degenerated CCs in AMD patients [111] (Figure 4C). Chymase and tryptase released from mast cells can activate proteolytic mediators such as matrix metalloproteinase-2, which is important for the remodeling of ECM components [112,113], like those found in Bruch’s membrane. The continuous growth and maturation of the neovascular membrane represent a balance between stimulatory and inhibitory signals of angiogenesis, inflammation, and proteolysis. Eventually, the balance shifts toward the regression of the neovascular vessels and the cessation of inflammation. This involutional stage is characterized by scarring and fibrosis [114]. The inflammatory component can vary according to the initiating pathology and contribute in varying degrees across the phases of CNV development. Similarly, the complement system or certain complement components can be differentially involved in the respective stages of CNV development [3], as further discussed in Section 6. Moreover, as emphasized in Section 5, it must be considered whether the relevant complement components promoting wAMD stem from the systemic reservoir or are produced locally.

## 5. Local Production of Complement Factors in the Retina

Tight junctions between the RPE cells constitute the outer blood-retina barrier (BRB) and, together with Bruch’s membrane, regulate the diffusion of inflammatory mediators and prevent the migration of immune cells from the blood to the retina (Figure 2A). This is also the role of the inner BRB formed by tight junctions between endothelial cells lining the retinal blood vessels (Figure 2A). In this way, the penetration of complement components from the blood into the retina is hindered [115]. On the other hand, the retina features the local production of complement proteins [56]. Besides providing the retina with the first-line defense, a local expression is also proposed to be a part of non-canonical functions, such as intracellular effects, which must be further investigated [59,116,117].

**Figure 5:**
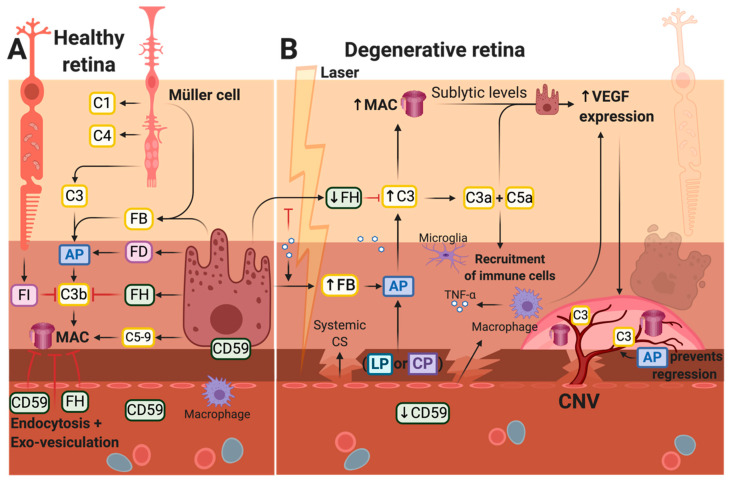
In vitro and in vivo studies indicate important roles for the AP, MAC, and the anaphylatoxins in CNV-formation. (**A**) In the healthy retina, complement activation and MAC-deposition in the RPE cells and CC are kept in check by inhibitors such as FH, CD59, and endocytosis [126]. (**B**) Shortly after laser-induction, the level of anaphylatoxins was found to increase [140], i.e., the complement system was activated. RPE cells in close proximity to the laser may degenerate directly due to coagulation necrosis, the following scar-formation, or CNV detaching the RPE from its source of nutrition. The activation of the complement system is pronounced in several cell layers of the outer retina and in the choroid. Therefore, it is not restricted to the specific regions shown above. AP, alternative pathway. CC, choriocapillaris. CNV, choroidal neovascularization. CP, classical pathway. FB, Factor B. FD, Factor D. FH, Factor H. FI, Factor I. LP, lectin pathway. MAC, membrane-attack complex. PR, photoreceptor. RPE, retinal pigment epithelium. TNF-α, tumor necrosis factor-alpha. VEGF, vascular endothelial growth factor.

The cell-type-specific expression of complement components in the retina was previously evaluated [59,118,119]. In the healthy retina of mice, the RPE produces factors belonging to the AP (i.e., FB, FD) [59,120] and the MAC (i.e., C5–C9) [59]. The RPE is also the main producer of FH in the retina in both humans [121] (Figure 4A) and mice [59] (Figure 5A). The same study observed that retinal neurons produce regulators such as FI and FP, while Müller cells are responsible for the main production of C1, C3, and C4 in mice [59] (Figure 5A). Furthermore, Natoli et al. identified retinal and subretinal macrophages to be the primary producer of local C3 in ARM and AMD as indicated by patient specimens and found that the local production of C3, but not serum C3, causally contributes to complement activation, yielding retinal degeneration in a model of photo-oxidative stress [119]. This indicates that the previous findings of C3 mRNA expression in isolated RPE-choroid and upregulated C3 expression after laser treatment in mice are primarily attributable to infiltrating macrophages [122]. Moreover, inflammatory cytokines produced by, e.g., macrophages can affect complement expression, as, e.g., TNF-α is found to stimulate the RPE-expression of FB and reduce that of FH in cultured mouse and human RPE cells (Figure 5B) [120,121]. Furthermore, microglia were found to be recruited to the outer retina and secrete C3 [119], and, in wAMD patients, microglia have been found in close contact with the CNV-complex [123] (Figure 4C).

Throughout life, oxidative damage will be exerted on the retina. This increase has been found to affect the expression of complement components in aging and AMD-diseased individuals [87,88,124]. For instance, the oxidized POS material can increase the synthesis of FB in human adult RPE (ARPE19) cells [120] and reduce that of FH in cultured mouse RPE cells [121]. This is consistent with the findings of reduced levels of FH in Bruch’s membrane, CC, and the choroid of AMD specimens compared to controls [125] (Figure 4C). Moreover, oxidative stress is connected to reduced surface expression of complement inhibitors such as CD59 as indicated for ARPE19 cells [124], and as RPE-bound CD59 is decreased in AMD specimens, e.g., above drusen [46] (Figure 4B,C). Thus, because of the higher FB levels and lower levels of FH and CD59, complement activation is favored in the presence of oxidative stress. This is evident in the eyes of AMD patients as (i) C3, FD, and FB are found to be increased in the interface between Bruch’s membrane and the choroid in both ARM and AMD patients [52] (Figure 4B,C), (ii) C3a, C3, FB, FBa, and FH are found with increased levels in the aqueous humor of wAMD patients [51,53] (Figure 4C), (iii) C3, FB, and FD are found to be significantly increased in the vitreous humor of both ARM and AMD patients [52] (Figure 4B,C), and (iv) activated FB, i.e., FBb used in the AP, is significantly elevated at the interface of Bruch’s membrane and the choroid in AMD eyes [52] (Figure 4C). The relevance of the AP in this complement activation was further substantiated, as (v) the total C3 protein, and not the C4a fragment present in the case of LP and/or CP activation, was found to be significantly higher at the interface of Bruch’s membrane and the choroid compared to the controls [52]. Furthermore, MAC accumulates with age in individuals without AMD, especially in the CC [15] (Figure 4A). However, compared to age-matched controls, (vi) MAC is more commonly deposited in ARM and AMD patients: In the outer choroid and hard drusen of ARM patients (Figure 4B), and in the choriocapillaris and possibly the RPE in wAMD patients [42] (Figure 4C).

The accumulation of sub-lytic levels of MAC stimulates the signaling pathways associated with tissue remodeling, inflammation, and angiogenesis [15]. Moreover, the inflammasome is stimulated, and the secretion of several elements, such as metalloproteases, reactive oxygen species, VEGF, and vitronectin (a MAC inhibitor), can be observed depending on the cells attacked by the complex [16,17,18]. One theory for this phenomenon is that sub-lytic levels of MAC are present in ARM, while lytic levels dominate in AMD [15]. Healthy host cells prevent MAC-deposition with FH, CD59, endocytosis, and exo-vesiculation [126,127]. The latter strategy possibly yields RPE blebs as cells try to shed membrane fragments (Figure 2B). However, when some individuals are unable to balance the accumulation of MAC and/or are unable to regulate the complement system, they can either become protected or prone to AMD. This balance is affected by factors such as the age and genetic variants of the individual [15]. For instance, in the retinas of aging mice, C1s, FB [120], FP, and FI expression was found to be enhanced, while FH expression seemed to be decreased [59], favoring complement activation.

Recently, the importance of local complement activation in the retina in ARM and AMD has become more evident, and the origins of the pathological complement components have been surveyed, as these compounds may stem from either the systemic complement reservoir produced by the liver [55] or by cells in the retina, such as RPE cells and/or immune cells [9,56,59,128]. Rohrer et al. found that the RPE-specific expression of FB is sufficient to drive complement activation, leading to RPE damage and CNV in FB knockout (KO) mice, whereas CNV lesions were significantly blunted in FB-KO mice [129]. This supports the findings that local intraocular complement activity is of greater importance for ARM and AMD pathogenesis compared to circulating complement components [130]. However, the authors also demonstrated that systemically recruited FB can promote CNV in FB KO mice treated with wild-type serum [129]. This indicates that the systemic contribution of complement components into the retina must be considered, as the local complement response can be amplified by components circulating in the blood and may contribute to the further progression of AMD.

In the healthy outer retina, tight junctions between RPE cells and the integrity of Bruch’s membrane maintain a non-inflammatory environment. The impermeability of a healthy Bruch’s membrane creates semi-independent compartments of complement regulation and activation in the retina and choroid, respectively. Thus, complement components primarily remain on either side of Bruch’s membrane based on the site of origin [115]. The permeability of Bruch’s membrane declines with age due to altered composition and deposit formation as well as the formation of drusen, as seen in ARM [115,131]. Eventually, the thickening of Bruch’s membrane influences the function and leads to changes in elasticity and hydraulic permeability. Loss of elasticity renders the membrane more brittle and susceptible to breaks, allowing neovascularization to grow into the subretinal space [132]. The barrier function of Bruch’s membrane is primarily compromised in wAMD patients [132,133,134]. Moreover, as retinal atrophy progresses, and, due to the high substance permeability of new vessels, systemic complement components can gain access to the diseased retina. In a recent study, the activation levels of systemic complements were found to be associated with AMD disease stages [49]. Patients with dAMD or active CNV had higher systemic complement activation levels compared with the controls, and this association was even more pronounced in patients with genetic variants associated with higher complement activation levels.

Taken together, these data indicate that both locally produced and systemically recruited complement factors can promote retinal pathology related to AMD. However, under normal conditions, locally derived complement factors predominate but are sensitive to complement components sequestered from the bloodstream when Bruch’s membrane and the BRB are breached. 

## 6. Complement Involved in Laser-Induced CNV Formation in Mice

The development of ARM is believed to be rather uniform in patients with either subtype of AMD. Complement components, especially C3, which is required for the complement system to work, may play a role in progression into AMD, such as the formation of CNV. Our current knowledge is derived from histopathology alongside aqueous and vitreous samples of CNV-affected eyes as well as information from the preclinical laser-induced CNV model. All complement pathways have been found to be relevant in the formation of laser-induced CNV in mice [135], but important roles are played by MAC [91,136,137], the AP [122,135,138], and the anaphylatoxins C3a and C5a [3,135,139,140] (Figure 5B).

Post-lesion, a reduction of both CD59 [137] and FH, as well as an upregulation of FB [138], are observed, thus, promoting activation of the complement and the formation of MAC. This is evident in the CNV complexes of mice, where C3 and MAC are found to accumulate [136] (Figure 5B). The overall activation may yield imbalances of anti-angiogenic and pro-angiogenic factors such as VEGF and PEDF [135], favoring neovascularization. This is substantiated as complement depletion, e.g., in mice treated with Cobra venom factor (that depletes for C3), reduced the levels of VEGF and CNV after laser-induction [136], and as reduced VEGF levels (as well as CNVs) were found in RPE-choroids after laser-induction of FB KO mice [122]. The main paths of complement activation toward neovascularization may involve infiltrating macrophages contributing to the increased local production of both C3 [119] and VEGF [110], as well as the stimulation of the RPE to secrete angiogenic factors such as VEGF, which can occur through sublytic levels of MAC [18,124]. Moreover, studies using ARPE-19 cells indicate a role of anaphylatoxins in stimulating angiogenesis [107,108] in addition to their role in the recruitment of immune cells.

CD59 is an essential inhibitor of MAC formation and affects CNV formation, as CD59a-deficient mice develop CNV earlier and more severely than control mice [136]. Moreover, intravitreal injections of recombinant CD59 (rCD59) yield less growth and reduced sizes of CNV [137,141]. The reduction of CNV in rCD59-treated mice may be a result of low MAC-deposition, resulting in less VEGF-secretion and, thus, increased apoptosis and decreased cell proliferation of the neovascularization complex in which the VEGF is a growth factor [141]. Moreover, mice deficient in C5 [138] and treated with C6-antibodies [91,136] or C9-antibodies [91] presented less MAC deposition and reduced CNV formation.

The AP is also involved in the laser-induced CNV as neovascularization is inhibited in mice treated with small interfering or short hairpin RNAs (shRNA) targeting FB [138,142] and FB-deficient mice [122,135]. This is substantiated by a reduction of VEGF in both FB shRNA-treated [142] and FB-deficient mice [122], possibly due to lower AP activity and, thus, lower MAC-deposition. Moreover, the amount of FB necessary for the AP to drive the laser-induced CNV is met by the local production of FB, as transgenic mice with a systemic deficiency of FB grew CNVs similar to wild-type mice [129]. Mice with only the AP (C1q^-/-^, MBL^-/-^) and FB-deficient mice (with no AP) were equally protected against retinal pathology following laser-induction, indicating that either the LP or CP has to be available to induce CNV [135]. Another study, however, suggested neither the LP nor the CP to be relevant, as C4-deficient mice (no LP or CP) evolved CNV equal to the control mice [138].

Since the CNV can be divided into an early phase, marked by injury and angiogenesis, and a late phase, comprising repair and/or fibrosis, Parsons et al. stressed the importance of considering these phases when seeking to treat wAMD with a complement therapy [3]. C3a, C5a, and their respective receptors (C3aR and C5aR) have been studied in the context of the initial formation of neovascularization, and conflicting results were found [140,143]. The anaphylatoxins, C3a and C5a, are generated shortly after laser-induction in mice [140] and are present long-term, such as 23 days post-lesion [3]. The antibody-mediated blockade of both C3a and C5a, as well as the genetic ablation of their receptors, were found to reduce CNV formation [140] possibly due to the ability of the anaphylatoxins to induce VEGF-secretion and lower PEDF-secretion from the RPE [107,108]. Conversely, this phenomenon may be connected to the capacity of these receptors to recruit immune cells. The anaphylatoxins and their receptors may also be involved in repair instead [144]. However, according to Parsons et al., late-stage CNV changes, such as repair, are unaffected by C3a-inhibition and C5a-inhibition [3]. Instead, this study found AP-inhibition to accelerate the regression and repair of CNV, indicating a possible strategy for CNV-regression therapy.

Poor et al. tested several approaches on different mouse strains and found a general difference in laser-induced CNV across strains [143]. For instance, C3-deficient and C5-deficient Jackson Laboratory (JAX) mice experienced increased CNV after laser-induction, while C3-deficient Taconic mice yielded reduced CNV. Moreover, these results indicated that a C5aR antagonist does not affect CNV, as had been reported by Nozaki et al. [140], and that CNV is unaffected by complement depletion through Cobra venom factor [143], as previously observed [136]. It should be noted that Poor et al. set out to investigate the reliability of the laser-induced mouse model and not to learn about the underlying pathological mechanisms of CNV. Their observations could be due to differences in the background genotype, rather than a lack of, e.g., C3. In these mice, the authors could only conclude that CNV can be formed in the absence of C3. The study indicates that studying the effect of complement components in CNV in mice provides challenges, and the mice models must be carefully chosen.

## 7. Current Treatment and Clinical Trials for Complement Inhibition in wAMD

Since the approval of the first injectable anti-VEGF drug in 2004, anti-angiogenic treatment has been the standard of care for wAMD patients to suppress CNV, reduce edema, and, thus, prevent vision loss [145]. To maintain a therapeutic concentration of the drug, the drug is injected monthly or several times a year depending on the agent and disease state. Several different drugs are currently used: (i) Ranibizumab (Lucentis^®^; Genentech), a Fab-fragment, (ii) Brolucizumab (Beovu^®^, Novartis), a single-chain variable fragment (ScFv) antibody, and (iii) Aflibercept (Eylea^®^, Regeneron Pharmaceuticals), which is a fusion protein of the VEGF receptor 1 and 2 binding domains fused to the constant region (Fc) of human immunoglobulin γ (IgG) 1 (VEGFR1/2-Fc). These three drugs are approved by the U.S. Food and Drug Administration (FDA) and the European Medicines Agency (EMA) for the treatment of wAMD. It is, however, evident that healing with this monotherapy is only partial. For example, Rofagha et al. studied the outcome of ~7-year Ranibizumab-treated patients and found only one-third of the outcomes to be good with a visual decline observed in half of the patients [146]. Thus, new treatment strategies are needed. Moreover, since complement activation is highly associated with wAMD, the inhibition of the complement system is evident.

The pathology of wAMD, wAMD-associated genetic variants, and possible side effects must be considered when choosing a complement target. For instance, knowledge about the specific complement deficiencies associated with certain infections or other health issues is necessary [147]. In the context of wAMD, all complement pathways seem relevant in preclinical trials of CNV formation [135], and important roles are played by MAC [91,136,137], the AP [135,138], and C3a and C5a [3,135,139,140], yielding several possible therapeutic targets. Clinical trials have tested complement therapies on both wAMD and dAMD [5,148,149,150,151,152,153,154], and efforts to evaluate the therapeutic effects are ongoing, but published data are sparse. In the following, we focus on the wAMD trials involving complement inhibition (Table 1). The complement targets are depicted in Figure 3.

C3 is a key target when broad inhibition of the complement system is required. C3 targeting will lower the activity of the complement cascade, as the AP will not amplify complement activation, healthy cells will not be tagged for degradation, the MAC and anaphylatoxins will not be formed, and immune cells, such as macrophages, will not be recruited, i.e., the pathologic production of VEGF will not be stimulated (Figure 4C and Figure 5C). C3-inhibition may, however, only be efficient if targeted locally, as C3 is present in high levels systemically [155]. The inhibition of C3 can be achieved via (i) the inhibition of C3-convertases, (ii) the addition of a regulator important in C3b-degradation, or (iii) a C3-inhibitor, such as the small molecule inhibitors POT-4 and APL-2. These two C3-inhibitors have been evaluated in patients with wAMD (Table 1). The intravitreal delivery of POT-4 was compared to Lucentis in patients with active wAMD in a phase I/II trial (NCT00473928, NCT01157065). POT-4 was unable to reduce central retinal thickness 12 weeks post-treatment, as seen with Lucentis [156]. The POT-4 derivative APL-2 was also evaluated in patients with active wAMD on anti-VEGF treatment (NCT02461771, NCT03465709). No data were published, and the phase I/II study was terminated. A phase II study in patients with dAMD (NCT02503332) indicated a decreased rate of GA progression following intravitreal injection of APL-2, but, paradoxically, a dose-dependent increase in the conversion to wAMD was observed, calling into question its use in wAMD patients [157]. 

Specific pathways could also be targeted to avoid complete inhibition of the complement system. Conflicting results were found concerning the essential role of either LP or CP in CNV-induction [135,138]. C2 and C4 are, however, both clear targets if one of these pathways is necessary for CNV. Since the AP is connected to AMD [3,81,158,159,160,161] and is essential for laser-induced CNV formation [135,138], AP-components are attractive targets. Moreover, the LP and CP can maintain host defense mechanisms with specific AP-targeting. Since the Y402H SNP in *FH* poses the greatest single genetic risk, FH is an interesting endogenous inhibitor that could be used therapeutically [3,122], as it lowers the activity of the AP through anti-C3-convertase activity, stimulates C3b-degradation, and exerts anti-oxidative effects toward the RPE [87]. The therapeutic potential of FH was demonstrated by Rohrer et al., as both intravenously administered CR2-FH, i.e., a recombinant form of FH, as well as locally injected adeno-associated viral (AAV) vectors encoding the CR2-FH attenuated laser-induced CNV in mice [122,162]. Moreover, FB, FD, and properdin are involved in the AP and are, thus, plausible targets. However, FH may be most favorable, as it spans a wider range as a protector of the retina [87,163,164]. Soluble complement receptor 1 (CR1) may also be considered as a possible therapeutic drug, as it prevents certain dysregulations of the AP fluid C3-convertase [165]. Furthermore, CR1 represses C3b and C6-accumulation [166], inhibits convertases, and affects C3b-degradation [167].

Targeting the terminal complement pathway and MAC-formation represents a further strategy that may lower VEGF-secretion and, thus, CNV formation [1,136,140]. Targets may be a component of the C5b–C9 complex, but the MAC-inhibitor CD59 could also be added. C5-inhibitors and sCD59 [137,168] have been tested in clinical trials for wAMD patients (Table 1). LFG316 is an IgG1 antibody that binds C5 and prevents its cleavage by the C5 convertase into C5a and C5b necessary for MAC-formation. Safety following intravitreal administration has been established in dAMD patients, but no effect on GA progression has been observed [169]. The results from a phase II trial in patients with active wAMD treated with anti-VEGF therapy are awaited (NCT01535950). Preliminary data (Gallemore RP et al. IOVS 2016, 57:ARVO E-Abstract 4986) did not provide evidence for an effect on the Best Corrected Visual Acuity (BCVA) or the rate of anti-VEGF retreatments. Another C5-inhibitor is the RNA aptamer Avacincaptad pegol (Zimura), which is being investigated clinically in several retinal disorders, including dAMD and wAMD. In a completed phase II trial (NCT03362190) on treatment-naïve wAMD patients, Zimura was combined with ranibizumab in different dosing groups. Its safety profile was encouraging, but its efficacy compared to anti-VEGF monotherapy could not be discerned, as no control group was included [170,171].

Intravitreal delivery of an AAV vector encoding human soluble CD59 (sCD59) is undergoing clinical evaluation (Table 1). The AAV-CAG-sCD59 vector (Hemera) was tested in dAMD patients with GA (NCT03144999) and alongside anti-VEGF therapy in treatment-naïve wAMD patients (NCT03585556). The results from these trials will provide insight into the potential of continuous complement inhibition and combination therapy. The use of combination therapies is regarded as a promising approach to supplement the presently used anti-VEGF treatment and also provides the rationale behind a novel bispecific decoy receptor fusion protein, IBI302, that was made to bind and inhibit VEGF, C3b, and C4b simultaneously [172] (Table 1). IBI302 has been tested in non-human primates [172] and is presently being tested in a Phase I trial (NCT03814291) on wAMD patients not receiving anti-VEGF therapy.

Since anaphylatoxins may stimulate angiogenesis [107,108], and because C3a-inhibition and C5a-inhibition were observed to reduce laser-induced CNV [3,139,140], both an anti-C3 strategy inhibiting the production of C3a and C3b (and all of their downstream components) and an anti-C5 strategy repressing both MAC and C5a may be efficient. More research is, however, needed concerning anaphylatoxins in the different stages of CNV [3] and the roles of macrophages as their facilitators [173].

## 8. Future Therapeutic Approach

The introduction of anti-VEGF therapies enabled the targeting of a central mediator of CNV development and revolutionized the treatment of wAMD. However, there is an evident need for optimized treatment protocols as (i) several patients were non-responders, (ii) the rescue was only partial, with a majority of patients experiencing continuous loss of visual function [146], (iii) the treatment burden is considerable with the current use of repeated intravitreal injections, and (iv) rare, but potentially sight-threatening, complications can occur.

As reviewed in this paper, several lines of evidence implicate the complement system in the pathogenesis of AMD, including the progression to, and continuous stimulation of, the neovascular stage (Figure 4). This suggests that complement targeting is a viable strategy. Disappointingly, proof-of-concept results in animal studies have not been readily translated into successful outcomes in clinical trials for either wAMD or dAMD. It must, however, be stressed that the trials conducted for wAMD are early phase studies that were primarily designed to evaluate safety issues, and the results from several trials are still awaited. Nonetheless, several challenges have been identified for the development of successful complement targeted therapies that are translatable to a clinical setting.

First, the ability of the retina to locally produce complement components capable of mounting or amplifying a complement attack is important to consider when designing complement therapeutics for treating AMD. In wAMD patients, the contributions from local and systemic complement regulators are blurred due to the breach of Bruch’s membrane (Figure 4C). That is, the pathological effects of the complement system in ARM possibly stem from complement expression of the retinal cells before the breach of Bruch’s membrane, while, in wAMD patients, the systemic complement system may also influence CNV formation and its perpetuation. Moreover, while the age-related thickening of Bruch’s membrane hinders most of the systemic complement components from entering the retina, C5a is still able to cross Bruch’s membrane due to its small size and net charge [115]. Thus, overactivation of the complement at Bruch’s membrane and the CC, as evidenced in AMD eyes by lower FH levels [125] and increased MAC-deposition [42], possibly yields C5a-induced inflammation and angiogenesis in both ARM and AMD [115].

If wAMD is a disease with systemic complement disturbances, systemic delivery could offer increased therapeutic benefits. For instance, the C5-inhibitor Eculizumab (Soliris) is used for systemic complement inhibition as an established treatment option for several diseases, such as atypical hemolytic uremic syndrome [174]. However, systemic inhibition of the complement system may not be the most efficient strategy for wAMD patients as (i) complement components can be produced by retinal cells and macrophages locally, (ii) frequent injections are required due to faster systemic clearance, (iii) side effects are more likely, and, (iv) because complement components, especially C3, are abundant in the blood, large amounts of complement inhibitors must be used to obtain a therapeutic effect.

Second, the extent to which the preclinical models recapitulate CNV formation in humans should be considered. The most prevalent model for CNV-induction in animals involves laser-induced breaks in Bruch’s membrane. While sharing features of human CNVs with the development of a mature fibrovascular membrane with subsequent regression, the resulting acute injury and inflammation do not correspond to the chronic degenerative and inflammatory pathology found in wAMD patients [175]. The evaluation of antibody-based therapies is additionally challenged by species specificity. The surrogate antibodies used in animal studies can vary in their therapeutic efficacy and pharmacokinetic properties [176].

Third, the use of intraocular delivery of complement regulators for treating wAMD should be considered carefully to allow the inhibitors to reach the site of the complement overactivation associated with wAMD. The current delivery strategy of drugs in retinal disorders relies on intravitreal injections routinely performed in office-based settings [177,178]. This is also the case for the clinical trials shown in Table 1. The diffusion of large molecules to the outer retina and RPE is limited by barriers, such as the inner limiting membrane [179,180] (Figure 2A). Both complete antibodies, such as bevacizumab [181,182], and antibody fragments, such as ranibizumab [183], have been demonstrated to reach the outer retina and choroid, but retinal penetration must be duly considered. Following injection, the agent is cleared from the vitreous and retina to systemic circulation, so continuous complement inhibition will require multiple injections as with current anti-VEGF therapies or necessitate the development of methods for long-term delivery. Such approaches could include controlled release systems or gene therapy. The latter is currently gaining momentum for the treatment of inherited retinal degenerations, and, as mentioned above, the AAV-vectored delivery of the complement inhibitor sCD59 is already under clinical evaluation in wAMD patients.

Subretinal injection of the therapeutic agent would deliver the agent immediately to the targeted tissue, potentially offering increased therapeutic efficacy. However, in contrast to intravitreal delivery, subretinal injections require vitreoretinal surgery, which has inherent complications and would involve the formation of sub-macular retinal detachment in the degenerated retina of wAMD patients. This would limit the use of subretinal injections to gene therapy or cell-based strategies utilizing a single injection for long-term management. In general, long-term therapies are preferred since they minimize the cost of treatment, lower the risk of side effects from injections, and are more convenient for patients. When delivering a transgene for continuous expression, the secretion of a soluble protein inhibitor is more optimal than CRISPR-based or RNAi-based strategies that only affect the target cells since several different cell types contribute to local disease progression.

A recent development involves suprachoroidal injections into the potential space between the sclera and the choroid with reports of more widespread distribution than subretinal injections and higher concentrations in the choroid and retina compared to intravitreal injections [184]. This administration route was approved for the delivery of triamcinolone to manage uveitic macular edema, but issues like systemic distribution and immune response must be considered, especially for gene therapeutic delivery. While this method is comparable to subretinal injections for outer retinal delivery without signs of extraocular transduction [185], increased local inflammation has also been observed with suprachoroidal injections. The systemic immune response was limited compared to intravitreal injection [186].

Fourth, the pathogenesis of wAMD and the role of the complement system, in particular, remain to be further elucidated. This includes the relative importance and temporal aspects of the complement system in AMD-development and CNV formation. This will help define optimal targets, the window of therapeutic efficacy, and the necessary duration of complement inhibition. Such insights could inform the rational design of clinical trials regarding the length of the treatment and follow-up as well as relevant patient populations. The outcome measures in the clinical trials must also be considered and defined, as the results will differ if evaluating CNV-reduction instead of a reduction in CNV growth [148]. In addition, patients refractive to anti-VEGF therapy may suffer a distinct subtype or harbor modulating genetic variants, and the inclusion of such patients in clinical trials of complement inhibition may, thus, provide misleading results.

Regarding the treatment window, a complement therapy aiming to treat ARM when the retina has not yet started to degenerate would benefit many patients in the long run. However, this treatment strategy would be expensive, and only a minority of patients with ARM develop sight-threatening changes in the macula. Late-stage disease, on the other hand, often involves a highly pro-inflammatory environment with irreversible loss of tissue, rendering most therapies including complement inhibitors ineffective, as suggested by the reviewed clinical trials. Presently, we cannot predict the progression and severity of disease in ARM patients, but, if this becomes possible, an anti-complement therapy could be beneficial in a subgroup of patients most likely to develop wAMD.

## 9. Conclusions 

To date, clinical trials testing complement therapies for wAMD have either been early phase studies or the results have remained unpublished. Nevertheless, only limited success has been observed in several trials, even though the agents seemed promising in theory and in vitro, as well as in preclinical trials. This apparent lack of success does not have one distinct cause but is most likely due to a combination of the chosen site of delivery, the concentration and the structure of the drug in the context of reaching the target tissue, the diffusion of the drug to the systemic circulation, the complement target, the stage of disease when treated, the skewed patient population, the targeting of only one pathway, and the outcome measures. Moreover, the physiological differences between studies done in vitro and preclinically, and the clinical trials may have been too large. However, surveying ongoing and future trials will be intriguing and relevant in the process of developing effective therapeutic strategies for these patients.

A complement inhibitor may be most efficient if delivered directly to the outer retina, where local disease develops and is continuously amplified. Inhibitors of the complement system can be recombinantly made or, for long-term management, encoded in viral vectors delivered to retinal target cells. The drug should be able to cross retinal cell layers and Bruch’s membrane but, at the same time, must remain locally at a therapeutic concentration, requiring a certain molecular structure and size of the inhibitor. It is questionable whether we know enough about the role of the complement system in wAMD to choose a suitable complement target and treatment window to obtain a therapeutic effect. Thus, more research is needed in this area, especially studies based on CNV specimens. Moreover, the complexity of wAMD pathology and the multitude of involved mediators and redundancy of implicated pathways, as well as the interactions of vascular and extravascular components in CNV formation, call into question whether a single target is suitable for the effective management of wAMD, thereby, providing an impetus for the development of combination therapies [187,188,189,190,191]. Ongoing trials of dual therapies suggest a trajectory involving combination therapies, and the results from these trials will illuminate the potential of this strategy. Complement inhibition will likely play a role as an adjuvant therapy to the targeting of VEGF and other factors in the context of combination gene therapy [188,190,191].

## Figures and Tables

**Table 1:** Complement therapies in clinical trials for patients with wAMD [5,148].

Target	Drug	Injection Site	Phase (Name), Trial Number	Status/Outcome
*C3*	**POT-4** (Potentia/Alcon) - Compstatin analog - Peptide	IVT	1 (AsAP) NCT00473928	**Completed**, Some clinical efficacy and no safety concerns
		IVT	2 (RACE) NCT01157065	**Completed**, Results from the phase I trial (AsAP) were not replicated
	**APL-2** (Apellis) - POT-4 derivative - Pegylated peptide	IVT	1 (AsAP II) NCT02461771	**Completed**, Unpublished
		IVT	1b/2 NCT03465709	**Terminated** (sufficient data were collected), Unpublished
*CD59*	Combination of: **AAVCAGsCD59** (Hemera) - Virus (AAV2) encoding soluble human CD59 and an **anti-VEGF treatment** - Bevacizumab (Avastin) - Ranibizumab (Lucentis) - Aflibercept (Eylea)	IVT	1 NCT03585556	**Ongoing**, Unpublished
*C5*	**LFG316** (Novartis Pharma AG) - Monoclonal human IgG1 ab	IVT	2 NCT01535950	**Completed**, Unpublished
		IV	2 NCT01624636	**Terminated**, Unpublished
	Combination of: **ARC1905** (Zimura, IVERIC) - RNA aptamer and **Ranibizumab** (Lucentis) - Humanized monoclonal Fab-fragment	IVT	1 NCT00709527	**Completed**, Well-tolerated and no evidence of acute toxicity
		IVT	2a NCT03362190	**Completed**, Generally well-tolerated. Studies were halted to focus on other studies (e.g., Zimura for GA)
*C3/C4*	**IBI302** - Bispecific decoy receptor fusion protein - Binds and inhibits VEGF, C3b + C4b simultaneously	IVT	1 NCT03814291	**Ongoing**

IVT, intravitreal. IV, intravenous. AAV, adeno-associated virus. VEGF, vascular endothelial growth factor. GA, geographic atrophy. C3, Complement component 3.

## Abbreviations

AAV	adeno-associated virus
AMD	age-related macular degeneration
AP	alternative pathway
ARM	age-related maculopathy
BRB	blood-retina barrier
C3	complement component 3 (same for C2, C4, etc.)
C3(H_2_O)Bb	fluid-phase C3-convertase
CC	choriocapillaris
CNV	choroidal neovascularization
CP	classical pathway
CR1	complement receptor 1
dAMD	dry AMD
EMA	European Medicines Agency
ECM	extracellular matrix
FDA	Food and Drug Administration
FB	factor B
FD	factor D
FH	factor H
FI	factor I
GA	geographic atrophy
GAG	glycosaminoglycans
GWAS	genome-wide association study
Ig	immunoglobulin
KO	knock-out
LP	lectin pathway
MAC	membrane-attack complex
MASP	MBL-associated serine protease
MBL	mannose-binding lectin
mRNA	messenger RNA
PEDF	pigment endothelial-derived factor
PR	photoreceptor
rCD59	recombinant CD59
RPE	retinal pigment epithelium
SNP	single nucleotide polymorphism
TNF-α	tumor necrosis factor-alpha
VEGF	vascular endothelial growth factor
wAMD	wet AMD
